# Effective situation-based delirium simulation training using flipped classroom approach to improve interprofessional collaborative practice competency: a mixed-methods study

**DOI:** 10.1186/s12909-022-03484-7

**Published:** 2022-05-27

**Authors:** Kiyoshi Shikino, Narumi Ide, Yoko Kubota, Itsuko Ishii, Shoichi Ito, Masatomi Ikusaka, Ikuko Sakai

**Affiliations:** 1 Department of General Medicine, Chiba University Hospital, 1-8-1 Chuo-ku Inohana, Chiba, Japan; 2 Interprofessional Education Research Center, Chiba University Graduate School of Nursing, Chiba, Japan; 3 Department of Nursing, Chiba University Hospital, Chiba, Japan; 4 Department of Pharmacy, Chiba University Hospital, Chiba, Japan; 5 Department of Medical Education Graduate School of Medicine, Chiba University, Chiba, Japan

**Keywords:** Flipped classroom, Interprofessional collaborative practice, Mixed method, Simulation

## Abstract

**Background:**

Interprofessional collaborative practice competency (ICPC) is key to providing safe, high-quality, accessible, patient-centred care. Effective delirium management, particularly, requires a multi-component intervention, including the use of interprofessional teams at care point. This research aims to investigate the effectiveness of the flipped classroom approach for improving ICPC in simulation-based delirium case management.

**Method:**

An embedded mixed-methods study was designed to investigate the effects of the flipped classroom approach on health professionals’ performance in delirium management. The study population comprised nine health professionals (three physicians, nurses, and pharmacists each). They used pre-class study materials about delirium management via a digital learning platform before a simulation case training session. A readiness assurance process test was conducted on key concepts, covered in the pre-class study material. Participants were randomly assigned to three teams, each of which included health professionals. Each team participated in a simulation case scenario. For the quantitative outcome measures, the Chiba Interprofessional Competency Scale (CICS29), a validated scale for measuring competencies of interprofessional practice, was used before, after, and three months after the educational intervention. The qualitative component consisted of a post-training questionnaire and semi-structured focused group interviews about the impact of the flipped classroom approach.

**Result:**

The CICS29 measured after the intervention and three months after was noted to be significantly higher than before the intervention. Three semi-structured focused group interviews were conducted (n=9), which, upon analysis revealed that the flipped classroom approach effected on four stages of Bloom's taxonomy level. A total of nine categories and 17 subcategories were identified corresponding to four levels of the revised Bloom’s taxonomy: remember (1), understand (12), apply (23), and analyse (3).

**Conclusion:**

The simulation-based skill training using flipped classroom approach can be an effective method for improving ICPC for health professionals. In this approach, an elevated level of cognitive activity is practiced in the Bloom’s taxonomy, and the participants worked on an application-based case simulation that promoted higher level learning and engagement in interprofessional collaborative practice. This approach also established a basic common language of delirium assessment and management, thus facilitating communication among health professionals and improving ICPC.

**Supplementary Information:**

The online version contains supplementary material available at 10.1186/s12909-022-03484-7.

## Background

Interprofessional collaborative practice competency (ICPC) is a key to the safe, high quality, accessible, patient-centred care [1]. In particular, effective delirium management requires a multi-component intervention, including the use of interprofessional teams at the point of care [2]. As an education model to improve interprofessional competency, the interprofessional-simulation experience can develop interprofessional competency [3, 4].

The flipped classroom model has emerged as an innovative solution to develop learner-centred learning [5, 6]. It is a learner-centred approach to teaching where the traditional class-time and self-study activities are reversed or “flipped.” [6–8] The course materials – reading materials, video lectures, and quizzes –are presented to the learners prior to attending in-person activities in the classroom and lower levels of learning objectives of the Bloom’s taxonomy are emphasized [9, 10]. The classroom’s physical and temporal space is reserved so the learners can apply, analyse, and evaluate (higher-order levels of learning objectives of the Bloom’s taxonomy) the newly learned material via in-person activities facilitated by a mentor and by collaboration with their peers [11, 12]. The active learning and differentiated instruction that the flipped classroom approach promotes, makes it effective in optimizing the use of live teaching time [13]. This results in a positive effect over the traditional teaching with respect to Bloom's higher order thinking and problem-solving skills [14–16].

Current evidence suggests that flipped classroom yields a significant improvement in health professionals’ learning than traditional teaching methods [17]. Additionally, some reports suggest the usefulness of flipped classrooms in the training of interprofessional collaborative practice competencies [18–20]. However, the educational effects on ICPC have not been well investigated in using this approach.

The objective of this research is to investigate the effectiveness of the flipped classroom approach for providing effective ICPC in simulation-based delirium case management.

## Methods

### Study design overview

Using a pragmatic approach, we employed an embedded mixed-method design that incorporated quantitative (questionnaires) and qualitative (focus groups) techniques [3, 21–25]. The design is a mixed methods approach in which qualitative data were collected following the intervention and analysed after the quantitative analysis [24]. This type of research study design capitalizes on quantitative and qualitative designs’ strengths while minimizing the shortcomings of each methodology. Furthermore, it allows the researchers to understand the experimental results better while incorporating the participants’ perspectives. The National Institutes of Health advises a mixed-method approach to conduct research that aims “to improve the quality and scientific power of data” and to better address the complexity of issues facing the health sciences today, including the health profession education [24, 25]. This study’s initial quantitative arm observed the Chiba Interprofessional Competency Scale (CICS29) scores, a validated scale for measuring competencies of interprofessional practice [26], before and after educational intervention. The qualitative data comprising health professionals’ perceptions were collected after the preliminary didactics experiment. We assumed that quantitative research alone could not sufficiently capture the participants’ cognitive processes, which influences the flipped classroom approach’s effectiveness for improving learning. Thus, we compared the revised Bloom’s taxonomy levels [8] of knowledge attained by the two groups of health professionals using the qualitative data (Supplement 1).

### Participants and context

The participants were selected using purposive sampling. There were two criteria for the participants to be included in this study: the first was that they were different health professionals primarily involved in delirium management and the second condition was that the participants were novice health professionals (having graduated less than five years ago as acute care health professions). This condition was selected given the potential relationship between the number of delirium management experienced. We recruited 12 health professionals working at Chiba University Hospital (graduated less than five years ago; four doctors, nurses, and pharmacists each) that satisfied the two conditions. Recruitment via email was conducted from January 2020 to February 2020. A power analysis using the G*power computer program [27] indicated that a sample of 12 people for each group would be needed to detect small effects (f = 0.25) with 80% power and alpha set at .05. Directors of health professional development centre, department of nurse, and department of pharmacy sent out recruitment emails to professionals in each of the group. Participants filled an information sheet and consent form. Moreover, they were also given the contact information of the researcher and a consent withdrawal form. They were informed that they could withdraw from the study at any point.

### Procedure and educational intervention

**Fig. 1:**
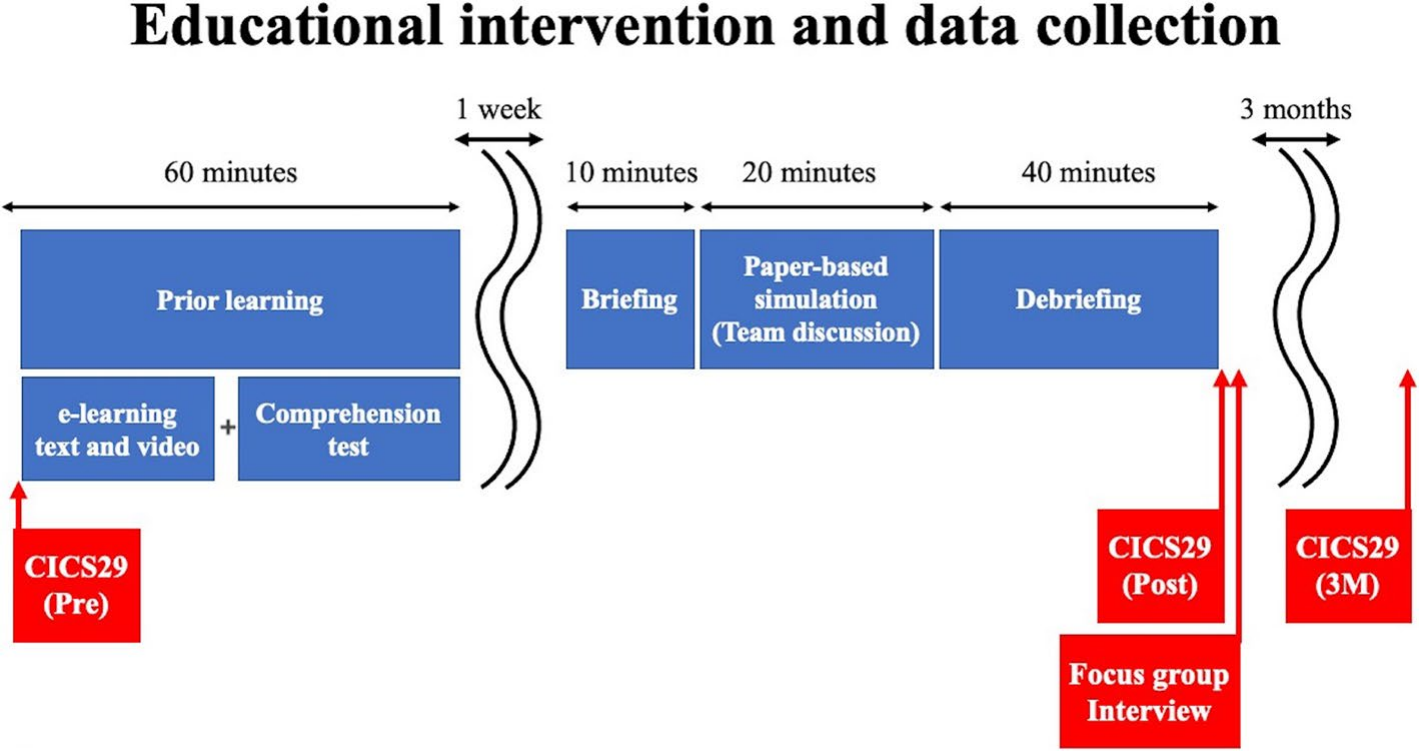
Education intervention and data collection

**Fig. 2:**
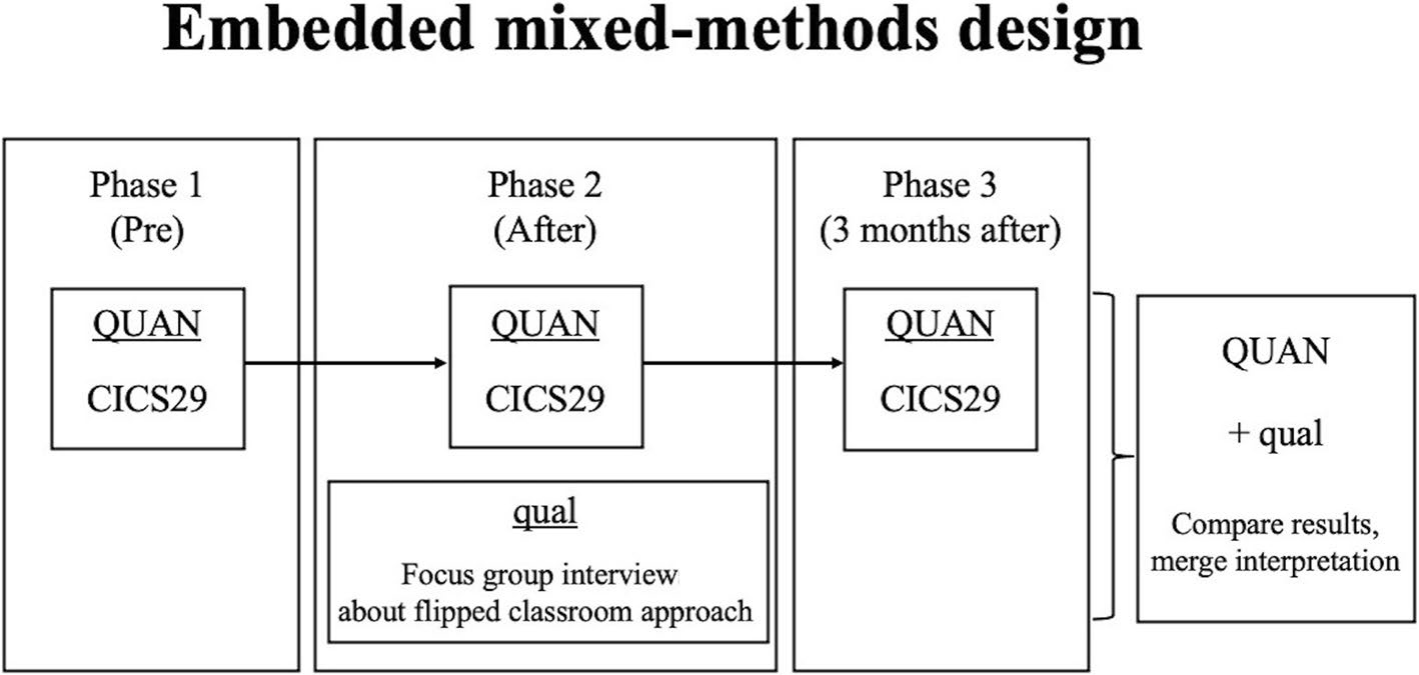
Embedded mixed-methods design

An embedded mixed-methods study was designed to investigate the effects of the flipped classroom approach on health professionals’ performance during delirium management (Figs. 1 and 2).

They studied the pre-class study materials about delirium management provided via a digital learning platform before a simulation case training session. The preliminary study materials included the definition and characteristics of delirium, subtypes, causal factors, and triggering drug agents. The preliminary study materials were developed based on the National Institute for Health and Care Excellence (NICE) guidelines for delirium care [28]. It also included an explanation of how to use the Japanese version of the 4A's Test for Delirium Screening [29], which was validated as an appropriate delirium screening tool. They underwent a readiness assurance process test comprising key concepts that they were expected to acquire from the pre-class study materials. A web-based comprehension test was administered in advance to ensure that everyone scored at least 80 points the first time (average 95 points).

Participants were randomly assigned to three group teams including each health profession. Each team participated in a simulation case scenario related to delirium (Supplement 2). Faculty members debriefed the health professionals about the assessment and management of delirium. For the quantitative outcome measures, the CICS29 measured pre-educational intervention, post-educational intervention, and the three months follow-up.

### Data collection

We collected two types of data: a pre / post / three months after questionnaire (quantitative, CICS29) and semi-structured focused group interviews (qualitative). The quantitative component comprised a post-training questionnaire. Focus group interviews were conducted immediately after the educational intervention.

1. *Pre / post / three months after questionnaire*

For the quantitative outcome measures, the CICS 29 (Supplement 3), a validated scale for measuring competencies of interprofessional practice, was used before, after, and three months after the educational intervention.

2. *Semi-structured focused group interviews*

A qualitative inquiry was conducted following the quantitative evaluation. A sample of nine health professionals were selected from the quantitative study participants [30]. After obtaining informed consent from them, we conducted interviews with three focus groups lasting about 60 minutes to minimize participants’ fatigue and regular workflow disruptions. Trained interviewers, who had experience in higher education in their respective countries and previously conducted educational research, asked open-ended questions about health professionals’ perception regarding the effectiveness of the flipped classroom approach on management of delirium in the paper-based simulation case. They asked about what went well and what did not in the educational session and the flipped classroom approach (Supplement 4). The responses of the focus groups were recorded and transcribed verbatim.

### Data Analysis

1. *Pre / post / 3 months after questionnaire*

To investigate the educational effectiveness of the flipped classroom approach for improving interprofessional collaborative practice competency in simulation-based delirium case management, we compared the pre-, post-, and three months after-evaluation CICS29 by the analysis of variance (ANOVA) and t-test. Statistical analyses were performed using IBM SPSS Statistics for Windows 26.0 (IBM Corp. Armonk, NY), with the level of significance set at P < 0.05.

2. *Semi-structured focused group interviews*

The transcripts were analysed using deductive content analysis, drawing upon the revised Bloom’s taxonomy as the coding frame, with cognitive process dimensions as the categories and sub-categories [31, 32]. Two authors did the initial coding of the focus group transcripts. One author independently read and coded all transcripts. Thereafter, they discussed, identified, and agreed on the coding of the descriptors. Following the coding, similar codes were grouped into categories and sub-categories, derived by an author as they emerged from the data. The categories and subcategories were regularly discussed on and reviewed for content by one author having experience in qualitative research to ensure credibility of the findings [12].

Concepts for each of the cognitive process dimensions in the revised Bloom’s taxonomy [9] were analysed, and the number of units of analysis for each concept was counted. The researchers then grouped similar codes into a theme and checked to see the dimension of the cognitive process to which it corresponded.

## Results

### Participants’ baseline characteristics

We received consent from all nine health professionals and were able to complete the quantitative and qualitative research. The study participants comprised nine health professionals (three physicians, nurses, and pharmacists, each; Table 1). Three participants did not provide their consent to the study and therefore did not participate. The participants had a median (interquartile range) work experience of three (range: 2-4) years, and five (55.6%) were women. Six participants attended the interprofessional education program when they were college students.

**Table 1:** Participant characteristics

ID	Group (No)	Work experience[*] (years)	Professions	Gender	IPE experience as a student
1	1	3	Nurse	Female	Yes
2	1	4	Pharmacist	Female	No
3	1	3	Physician	Male	No
4	2	3	Nurse	Female	No
5	2	3	Pharmacist	Male	Yes
6	2	3	Physician	Male	Yes
7	3	3	Nurse	Male	No
8	3	3	Pharmacist	Female	Yes
9	3	2	Physician	Female	Yes

Median length of work experience 3 years (range: 2-4 years)

### Quantitative main outcomes and measures

The CICS29 measurements after the intervention and after three months were significantly higher than those before the intervention (105.8 ± 10.1 vs 120.9 ± 9.5, *p*=0.003; 105.8 ± 10.1 vs 115.8 ± 9.4, *p*=0.047, respectively) (Table 2).

**Table 2:** Changes in Chiba Interprofessional Competency Scale (CICS29)

**Variable**	**Pre-Session**	**Post-Session**	**3-Month Post-Session**
I : Attitudes and beliefs as a professional	21.3±2.1	24.9±3.2^*^	24.2±2.2^*^
II : Team management skills	17.0±2.5	21.0±2.0^*^	20.0±2.2^*^
III : Actions for accomplishing team goals	17.1±2.1	21.6±2.1^*^	19.2±2.2
IV : Providing care that respects patients	21.0±1.4	21.6±1.7	21.2±1.6
V : Attitudes and behavior that improve team cohesion	14.3±2.2	17.7±1.7^*^	17.3±1.1^*^
VI : Fulfilling oneʼs role as a professional	15.2±1.7	16.7±2.0	15.8±1.6
Total	106.0±9.4	123.3±10.5^*^	117.8±8.9^*^

An independent-sample Kruskal-Wallis test based on the distribution of data. The maximum score for CICS29 was 145 (I: 30, II: 25, III: 25, IV: 25, V: 20, VI: 20)

*Significant difference from pre-session scores, *P* < .05

### Content analysis

The educational flipped classroom method’s effect on the learners’ cognitive processes between intervention and control groups was explored in this study. Thematic saturation was reached after analysing transcripts from three focus groups. The absolute frequencies of the codes for each cognitive process dimension for our data are presented in Table 3. A total of nine categories and 17 subcategories were identified corresponding to four levels of the revised Bloom’s taxonomy[7]: remember, understand, apply, analyse (Table 3). The most frequent subcategory by the number of codes was “apply” in the flipped classroom approach.

**Table 3:** Absolute frequencies of codes for each category and subcategory.

Flipped classroom approach				
Cognitive process levels from the revised Bloom’s Taxonomy	Category	Subcategory	Quotes	
Analyse (3)[*]	Quality improvement (2)	Quality improvement of delirium management (2)	‘*I think the impact of the preliminary study on management is that the preliminary study materials have helped us see more clearly how to manage delirium from the perspective of nurses and pharmacists to improve the quality of the patient.*	
	Self-analysis (1)	Metacognition (1)	*‘I was able to recognize objectively through this simulation that I do not fully understand the three categories of direct, induced, and preparatory factors.’*	
Apply (23)	Interprofessional team collaboration (9)	Contribution as professional roles (5)	‘*I had an idea of the factors that lead to delirium in my preliminary study, so I think I was able to directly ask myself how I should treat this patient in such a case.*’	
		Team building (2)	*‘The team clearly understood the team's goals and the teamwork was smoother as a result, since everyone knew the common knowledge about dealing with delirium from the preliminary study.’*	
		Utilization of professional perspectives (1)	*‘From the nurses' perspective, the patient was able to assess his activities of daily living at home, which had been fine in the past, but had not been possible after hospitalization.’*	
		Gathering information through interprofessional collaboration (1)	*‘I thought delirium was likely to occur at the time of admission, such as the patient's background. I was able to realize this time that when we see a patient like this, what kind of information, if collected by health professions, we can respond quickly.’*	
	Problem solving (10)	Applying knowledge of delirium response (5)	*‘I usually deal only with drugs, so I was glad that I knew in the preliminary learning that there are factors that contribute to this kind of delirium, and I was able to use that knowledge in this simulation.’*	
		Categorizing delirium factors (3)	*‘By doing my preliminary learning, I was able to identify and categorize the factors of delirium that needed to be addressed. I believe this is what I was able to use in the simulation, what I had learned in the preliminary study.’*	
		Decision making as professional roles (2)	*‘I learned that I can score delirium to make a diagnosis and consider whether or not it is delirium, making it easier to understand how to determine delirium from my profession's role.’*	
	Positiveness (3)	Motivation (1)	‘*Since it is assumed that the knowledge learned in the preliminary study will be used in this simulation, I found the flipped classroom approach to be more practice-oriented, and therefore, I felt that I would learn more or actually become more motivated to learn on my own than in a lecture.*’	
		Self-explanation (1)	*‘I end up being passive in my learning in a traditional lecture. This time, I had an opportunity to speak on my own, so I thought what I learned was more likely to leave a lasting impression.’*	
		Active participation (1)	*‘I was able to actively add to the discussion about patient information that I also knew.’*	
	Translational simulation (1)	Discussions in line with actual clinical practice (1)	‘*Since I knew what factors lead to delirium in the preliminary study, I was able to learn how I would respond in the case of an actual patient.*’	
Understand (12)	Assessment and diagnosis (9)	Understanding delirium assessment approach (6)	*‘I think it is good to have a true objective and unbiased measure of delirium as a common terminology, rather than just thinking it is delirium somehow, since the scoring of delirium can now be assessed with the objective approach.’*	
		Understanding diagnostic process (3)	*‘Through my preliminary learning, I learned delirium scoring, which helped me to understand the diagnostic process of delirium.’*	
	Common terminology (3)	Using common language of each profession (3)	‘*I was able to learn a common language through the preliminaries. In this regard, I found it good that the scoring of delirium is an objective number, so I can take a more objective view of delirium and share it with other health professionals, rather than just thinking that it is just delirium.*’	
Remember (1)	Memory retention (1)	Memory retention (1)	*‘I thought that with lectures, time would end up being passive. Today, I had a chance to speak on my own, so I thought it would be easier to leave a lasting impression.’*	

^*^( ) number of codes

The 39 codes generated from the FGI verbatim transcripts were aggregated by similar content to generate nine subcategories. The semantic content of the subcategories was further classified into four categories of similar content based on the participants' perceptions of the program's effects, and these categories were then arranged by process level according to the revised Bloom's taxonomy classification.

The revised Bloom's classification *Remember* (1) generated the category *Memory Retention*.

The revised Bloom's Classification *Understanding* (12) generated the subcategories *Understanding the delirium assessment approach*, *Understanding the diagnostic process*, and *Using the common language of each profession*, which were aggregated into the category, *Assessment and diagnosis*.

*‘I think it is good to have a true objective and unbiased measure of delirium as a common terminology, rather than just thinking it is delirium somehow, since the scoring of delirium can now be assessed with the objective approach.’(ID = 3)*

Further, the subcategory *Using the common language of each profession* was created under the category, *Common terminology*.

.‘*I was able to learn a common language through the preliminaries. In this regard, I found it good that the scoring of delirium is an objective number, so I can take a more objective view of delirium and share it with other health professionals, rather than just thinking that it is just delirium.*’ (ID = 4)

The subcategories *Applying knowledge of delirium response*, *Categorizing delirium factors*, and *Decision making as professional roles* were classified under the category of *Problem solving*.

‘*By doing my preliminary learning, I was able to identify and categorise the factors of delirium that needed to be addressed. I believe this is what I was able to use in the simulation, what I had learned in the preliminary study.*’ (ID = 9)

The subcategories, *Motivation*, *Self-explanation*, and *Active participation* were classified into the category, *Positiveness*.

‘*Since it is assumed that the knowledge learned in the preliminary study will be used in this simulation, I found the flipped classroom approach to be more practice-oriented, and therefore, I felt that I would learn more or actually become more motivated to learn on my own than in a lecture.*’ (ID = 8)

Furthermore, the subcategory, *Discussions in line with actual clinical practice* was created under the category, *Translational simulation*.

‘*Since I knew what factors lead to delirium in the preliminary study, I was able to learn how I would respond in the case of an actual patient.*’ (ID = 8)

The revised Bloom's Classification Application (23) generated the subcategories, *Contribution as professional roles*, *Team building*, *Utilization of professional perspectives*, *Gathering information through interprofessional collaboration*, which were aggregated into the category of ‘interprofessional team collaboration’.

‘*I had an idea of the factors that lead to delirium in my preliminary study, so I think I was able to directly ask myself how I should treat this patient in such a case.*’ (ID = 7)

The subcategories, *Applying knowledge of delirium response*, *Categorizing delirium factors*, and *Decision making as professional roles* were generated in the category *Problem solving*.

*‘I usually deal only with drugs, so I was glad that I knew in the preliminary learning that there are factors that contribute to this kind of delirium, and I was able to use that knowledge in this simulation.’(ID=6)*

The subcategories, *Motivation*, *Self-explanation* and *Active participation* were generated in the category, *Positiveness*.

*‘Since it is assumed that the knowledge learned in the preliminary study will be used in this simulation, I found the flipped classroom approach to be more practice-oriented, and therefore, I felt that I would learn more or actually become more motivated to learn on my own than in a lecture.’(ID=2)*

The subcategory, *Discussions in line with actual clinical practice* was generated under the category, *Translational simulation*.

*‘Since I knew what factors lead to delirium in the preliminary study, I was able to learn how I would respond in the case of an actual patient.’*

In the revised Bloom's classification analysis (3), the subcategory, *Quality improvement of delirium management* was moved to the category, *Quality improvement*.

‘*I think the impact of the preliminary study on management is that the preliminary study materials have helped us see more clearly how to manage delirium from the perspective of nurses and pharmacists to improve the quality of the patient.* (ID = 1)

The subcategory *Metacognition* was created in the category, *Self-analysis*.

*‘I was able to recognize objectively through this simulation that I do not fully understand the three categories of direct, induced, and preparatory factors.’(ID=7)*

## Discussion

This study suggests that the flipped classroom approach can improve ICPC not only immediately after educational intervention but also three months post the intervention, and the effect may be long-lasting. Additionally, the content analysis showed that the flipped classroom approach affected the cognitive process level of the revised Bloom’s taxonomy from ‘remember; to ‘analyse’. This may be because the flipped classroom approach may enable higher levels of cognitive activity according to the revised Bloom’s taxonomy[9, 10, 17].

The flipped classroom approach may offer advantages for interprofessional collaboration in education for teaching of the basic knowledge and understanding of delirium assessment and management approach. This knowledge and understanding can be acquired during early interprofessional education stages, for example, medical school using the flipped classroom teaching approach[33].

This content analysis suggested that a ‘common language’ was analysed as a strength of the flipped classroom in interprofessional collaboration practice and one of the barriers to its development is the lack of the common language of each profession group[34, 35]. The flipped classroom approach established a basic common language of delirium assessment and management, thus facilitating communication among health professionals and improving ICPC. Common language is involved in the items addressed in domain I: Attitudes and beliefs as a professional. The fact that this domain maintained the improvement after three months, also suggests the effectiveness of combining the flipped classroom method with simulation education.

In this research, content analysis identified a total of nine categories and 17 subcategories corresponding to four levels of the revised Bloom’s taxonomy; and the most frequent subcategory by the number of codes was *apply*in the flipped classroom approach. A study that evaluated the educational effects of flipped classroom method using a modified version of Bloom's Taxonomy also found equivalent results[36]. Mastering a common language, understanding basic knowledge, and practicing applications in simulations may be the factors that have been effective.

In the current study, the CICS29, showing ICPC, remained effective even three months after the pre-educational intervention. The results of the content analysis also extracted memory retention as an advantage of flipped classroom approach. Some evidence showed that flipped classroom approach is helpful in improving learner’s long-term memory retention[37, 38]. This approach is considered to promote long-term retention in learners, related to interprofessional collaborative practice competency, by applying the knowledge acquired through advance learning.

Furthermore, the ‘interprofessional team collaboration’ extracted in the category corresponds exactly to Domain VI: Fulfilling one's role as a professional in CICS29. The quantitative data showed no significant differences, however, consistent with all other domains, the high values immediately after the educational intervention are constant with the data.

However, in Table 2 outcomes, it is interesting to note, that the 3-month post session is not only still showing its effect, but also a decline from the that immediately after the intervention session. This decline is nevertheless, better than pre-training. This could demonstrate a decline in education/knowledge, which is common with time unless the healthcare provider has continuous education or experience. Therefore, continuous education would also be necessary for continued success even with the flipped classroom approach.

Simulation-based education can help healthcare professionals achieve higher levels of competence and safer care[3, 39]. Additionally, some studies have shown high educational effectiveness by using simulation education with flipped classrooms[39]. The incorporation of patient simulation into the flipped classroom approach could improve learners' long-term knowledge retention of disease and enhance their confidence in caring for these patients in their internship[40]. This may contribute to the improvement of ICPC.

### Limitations

There are some potential limitations of the current study. First, although introducing flipped classroom approach was found to improve ICPC, comparisons with other teaching methods, such as traditional classroom teaching style, were not conducted. Second, there were only nine eligible participants. The anticipated sample size required 12 participants, but three were unable to give consent. The fact that three of the 12 participants dropped out may have been due to the load of data collection. Third, since the study was conducted by health professionals at a single facility, which is a university hospital, further validation is needed to determine whether the results can be generalised to other facilities. Fourth, the study’s participants may be highly motivated to learn at the time they consented to the study although recruiters used purposive sampling. We also have to consider purposive sampling and the healthcare providers’ IPE experiences as confounders as not all had same experience. One of the main challenges associated with flipped classroom approach is that it requires learners’ self-motivation of self-directed learning for their education. For real-world applications, consideration will have to start with efforts to improve motivation in the first place. Fifth, various confounding factors, such as actual work, self-study, participation in workshops, among others during the three-month period may have influenced on the results of three months after the educational intervention, which were not investigated in the current study. In particular, it has not been possible to rigorously analyse the extent to which the effects of this time were due to simulation education and the extent to which they were due to flipped classroom approach.

## Conclusions

The simulation-based skill training using flipped classroom approach can be an effective method for improving ICPC for health professionals.

## Availability of data materials

The raw dataset supporting the conclusions of this article is available from the corresponding author upon request.

## Supplementary Information

**Additional file 1: Supplement 1.** Bloom's taxonomy.

**Additional file 2: Supplement 2.** Case scenario.

**Additional file 3: Supplement 3.** The 29 items of Chiba Interprofessional Competency Scale (CICS29).

**Additional file 4: Supplement 4.** Interview guide.
