# Supplementary figures and images for: Effective situation-based delirium simulation training using flipped classroom approach to improve interprofessional collaborative practice competency: a mixed-methods study
Source: BMC Med Educ. 2022 May 27;22:408. doi: 10.1186/s12909-022-03484-7 (PMC9137075; doi:10.1186/s12909-022-03484-7)

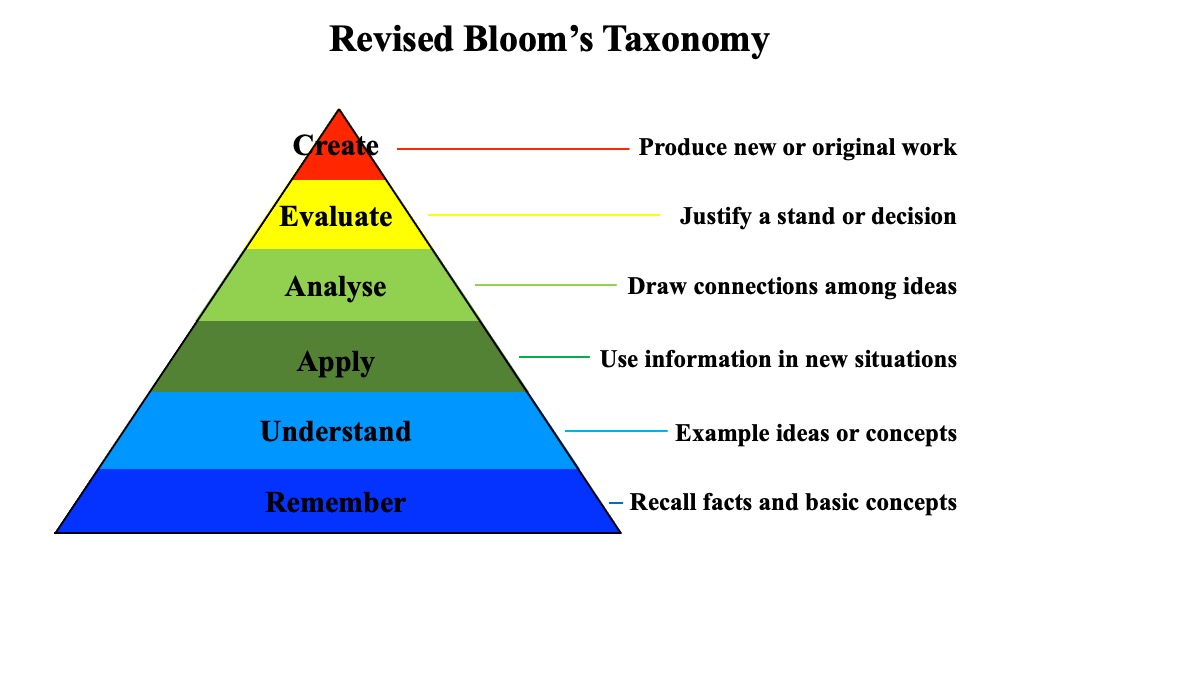

Supplement: Supplementary file 1 — Additional file 1: Supplement 1. Bloom's taxonomy. [file 12909_2022_3484_MOESM1_ESM.jpg]
