# Supplementary material for: Effective situation-based delirium simulation training using flipped classroom approach to improve interprofessional collaborative practice competency: a mixed-methods study
Source: BMC Med Educ. 2022 May 27;22:408. doi: 10.1186/s12909-022-03484-7 (PMC9137075; doi:10.1186/s12909-022-03484-7)
Supplement: Supplementary file 2 — Additional file 2: Supplement 2. Case scenario. [file 12909_2022_3484_MOESM2_ESM.docx]

**Supplement 2. Case scenario**

1. Role

| Physician | On night ward duty |
| --- | --- |
| Nurse | In charge of the patient |
| Pharmacist | On night duty |

1. Methos

Face-to-face, and paper-based simulation case

1. Patient information

| Identification | 82-year-old man |
| --- | --- |
| Diagnosis on admission | Bacterial pneumonia, chronic heart failure, hypertension |
| Hospital setting | University hospital, Department of general internal medicine |
| Medications | Amlodipine 5mg 2 t once a day  Telmisartan 80 mg 1 t once a day  Furosemide 40 mg 2 t twice a day  Aspirin 100 mg 2 t once a day  Famotidine 20 mg 1 t twice a day  Vildagliptin 50 mg 2 t twice a day |
| Past medical history | Hypertension, chronic heart failure, diabetes, and insomnia. |
| Social history | Smoking: 1 pack/day  Alcohol: 2 glass of sake every day |
| Family structure | Live alone. Eldest son and his wife live in the same city (about 20 minutes by car). |

1. Admission clinical course

| On the 1st day in the hospital (admission day) | The patient was seen at a clinic for fever and respiratory distress. Bacterial pneumonia and chronic heart failure were suspected, and the patient was referred to our department of general internal medicine (GIM) for hospitalization. The patient was admitted to a private bed in GIM ward at 17:00 on the same day for treatment of bacterial pneumonia and chronic heart failure. After securing a peripheral venous line, fluid administration (1000 mL/day), antibacterial agents (sodium sulbactam and sodium ampicillin 6 g/day, twice daily), and oxygen (2 L nasal) were started. |
| --- | --- |
| On the 2nd day in the hospital | On the 2nd day of hospitalization, at approximately 8:00 p.m., a nurse discovers during rounds that the patient was attempting to self-extract a peripheral IV line. |

1. Physical examination

| Vital sign | BP 142/72 mmHg, BT 37.8℃, HR 102/min, RR 14/min, O2 Sat: 97%(2L nasal) |
| --- | --- |
| Neck | JVD (-) |
| Lung | Coarse crackle at right lung field |
| Cardiac | S1(+), S2(+), S3(-), S4(-), murmurs(-) |
| Extremities: | Bilateral legs edema (+) |

1. Questions from the facilitator (initiation)

"Next we move on to the case scenario, where we are divided into teams of three. Teams consist of a physician, nurse, and pharmacist. Scenario cases will be presented in each room, and teams will work on their assignments. A debriefing will be given by the facilitator afterwards. Please be confident in your approach as a health profession."
