# Supplementary material for: Effective situation-based delirium simulation training using flipped classroom approach to improve interprofessional collaborative practice competency: a mixed-methods study
Source: BMC Med Educ. 2022 May 27;22:408. doi: 10.1186/s12909-022-03484-7 (PMC9137075; doi:10.1186/s12909-022-03484-7)
Supplement: Supplementary file 3 — Additional file 3: Supplement 3. The 29 items of Chiba Interprofessional Competency Scale (CICS29). [file 12909_2022_3484_MOESM3_ESM.docx]

**Supplement 3. The 29 items of Chiba Interprofessional Competency Scale (CICS29)**

| Domain Ⅰ: Attitudes and beliefs as a professional | |
| --- | --- |
| 1 | I constantly strive to improve my performance |
| 2 | I always reflect on the care that I have provided |
| 3 | I strive to be a professional |
| 4 | I practice evidence-based care |
| 5 | I am able to explain the basis for care to anyone |
| 6 | I am able to apply updated expert knowledge to actual practice |
| Domain Ⅱ: Team management skills | |
| 7 | I understand the scope and limits of my team members’ work |
| 8 | I respect my team members’ busy schedules and work pace |
| 9 | I cooperate with my team members to try to solve the problem when the team is not functioning well |
| 10 | I reconcile conflicts among team members |
| 11 | I know when problems within the team are likely to arise |
| Domain Ⅲ: Actions for accomplishing team goals | |
| 12 | I am able to explain the results of my team’s initiatives |
| 13 | I am able to adjust my practices to achieve the team’s objectives |
| 14 | I am able to coordinate the opinions of myself and my team members in light of the team’s objective |
| 15 | I provide necessary support to my team members depending on their professional competency |
| 16 | I am able to evaluate whether the team is operating well objectively |
| Domain Ⅳ: Providing care that respects patients | |
| 17 | I respect not only the wishes of the patient but also those of their family |
| 18 | I keep patient independence in mind when providing care |
| 19 | I interact with patients to help them make their own decisions |
| 20 | I change my manner of interacting with patients on the basis of their characteristics and situations |
| 21 | I seek the best way to care for patients |
| Domain Ⅴ: Attitudes and behaviours that improve team cohesion | |
| 22 | I consciously create opportunities for communication with other professionals |
| 23 | I discuss ideal patient care daily with other professionals |
| 24 | I try to create a suitable atmosphere during meetings wherein it is easy for other professionals to speak |
| 25 | I strive daily to create good interpersonal relations between professionals |
| Domain Ⅵ: v | |
| 26 | I am able to express opinions in front of other professionals on the basis of my expert knowledge |
| 27 | I fulfil my professional role as required by my team |
| 28 | I understand the scope of what can be accomplished by professional expertise and skills |
| 29 | I am able to state my opinions when necessary from the viewpoint of my professional expertise, even if doing |

Chiba Interprofessional Competency Scale (CICS29) is a scale to measure the ability to practice multidisciplinary collaboration (validated).^*^

By answering 29 questions, it is possible to assess collaboration skills categorized in 6 areas.

If you would like to use CICS29, please email us at Professor Ikuko Sakai, Graduate School of Nursing, Chiba University, Chiba, Japan.

e-mail: ikusakai@faculty.chiba-u.jp

^*^Sakai I, et al. J Interprof Care. 2017;31(1):59-65.
