# Supplementary material for: Effective situation-based delirium simulation training using flipped classroom approach to improve interprofessional collaborative practice competency: a mixed-methods study
Source: BMC Med Educ. 2022 May 27;22:408. doi: 10.1186/s12909-022-03484-7 (PMC9137075; doi:10.1186/s12909-022-03484-7)
Supplement: Supplementary file 4 — Additional file 4: Supplement 4. Interview guide. [file 12909_2022_3484_MOESM4_ESM.docx]

**Supplement 1. Interview guide**

1. General briefing
2. Interview content

Q1: "We learned a common language about physical assessment such as 4AT-J in this flipped classroom. What do you think is the significance of attending the seminar having done the preparation?"

Q2: "What are some of the points where you were able to use your prior learning in assessment/diagnosis?"

Q3: "Please tell me the points you were able to use the prior learning in your response at the onset of the disease."

Q4: "Please indicate the points where you were able to use your prior knowledge in preventative intervention."

1. Closing remark
